# Complete Genome Sequence of *Pseudomonas* sp. Strain SGAir0191, Isolated from Tropical Air Collected in Singapore

**DOI:** 10.1128/MRA.00617-19

**Published:** 2019-08-22

**Authors:** Anthony Wong, Ana Carolina M. Junqueira, Ankur Chaturvedi, Akira Uchida, Rikky W. Purbojati, James N. I. Houghton, Caroline Chénard, Megan E. Clare, Kavita K. Kushwaha, Alexander Putra, Nicolas E. Gaultier, Balakrishnan N. V. Premkrishnan, Vineeth Kodengil Vettath, Cassie E. Heinle, Daniela I. Drautz-Moses, Stephan C. Schuster

**Affiliations:** a Singapore Centre for Environmental Life Sciences Engineering, Nanyang Technological University, Singapore; b Departamento de Genética, Instituto de Biologia, Universidade Federal do Rio de Janeiro, Rio de Janeiro, Brazil; University of Arizona

## Abstract

*Pseudomonas* sp. strain SGAir0191 was isolated from an air sample collected in Singapore, and its genome was sequenced using a combination of long and short reads to generate a high-quality genome assembly. The complete genome is approximately 5.07 Mb with 4,370 protein-coding genes, 19 rRNAs, and 73 tRNAs.

## ANNOUNCEMENT

Species belonging to the genus *Pseudomonas* are Gram-negative rod members of the Gammaproteobacteria (1). They were originally isolated from rice and cereals (2) and have been found in insect hosts as a key mediator in the breakdown of caffeine inside their guts (3). While rare, some species of *Pseudomonas* have been found in secondary surgical and traumatic wound infections (4–6). The genus *Pseudomonas* is known for its large and diverse grouping, and its taxonomy is still being defined, with more than 70 species described so far (7).

*Pseudomonas* sp. strain SGAir0191 was isolated from an air sample collected in Singapore (global position system coordinates, 1.350°N, 103.68°E) utilizing the Andersen single-stage impactor (SKC BioStage, USA). Air was impacted onto M9 minimal salt medium (MP Biomedicals, Singapore) supplemented with 10 g/liter sodium carboxymethylcellulose (Sigma-Aldrich, Singapore). Further isolation was carried out by culturing on Trypticase soy agar (Becton, Dickinson, USA) at 30°C. Prior to DNA extraction, a single colony was cultured in lysogeny broth (Merck, USA) at 30°C overnight. Genomic DNA was extracted using the Wizard genomic DNA purification kit (Promega, USA) per the manufacturer’s protocol. The sequencing library was constructed with the SMRTbell template prep kit 1.0 (Pacific Biosciences, USA), and single-molecule real-time (SMRT) sequencing was performed on the RS II platform (Pacific Biosciences). In addition, whole-genome shotgun libraries were constructed with the TruSeq Nano DNA library kit and sequenced on the MiSeq platform (Illumina, USA).

Subsequent analysis was performed using default software parameters unless otherwise specified. A total of 86,946 PacBio subreads with an *N*_50_ length of 17,551 bp were used for *de novo* assembly with Hierarchical Genome Assembly Process version 3 implemented in the PacBio SMRT Analysis package version 2.3.0 (8). The assembly was polished with Quiver (8) and error corrected using 848,790 Illumina paired-end reads (300 bp) with Pilon version 1.16 (9). The consensus assembly generated a circularized chromosome of 5,071,227 bp with an average coverage of 183.94-fold and a mean G+C content of 61.43%. Circularity was confirmed using Circlator version 1.1.4 (10).

Taxonomic identification was performed using Phyla-AMPHORA (11) using MarkerScanner.pl with added “-DNA” flag and MarkerAlignTrim.pl with the options “-WithReference” and “-OutputFormat phylip.” Phylotyping.pl was run with default parameters, showing that SGAir0191 has 96.9% of markers matching the genus *Pseudomonas*. Species evaluation was also performed with Microbial Species Identifier based on a genome-wide average nucleotide identity (ANI) run using ANICalculator with default parameters against a database of 6,387 bacterial RefSeq genomes created using text filter for type, synonym type, and proxytype and subsequently getorf with the -find 3 option (12). Results showed 94.8% identity to Pseudomonas fulva NBRC 16637 (= DSM 17717) with an alignment fraction value of 56%. SGAir0191 was added under the parent node of 136845, which belongs to the Pseudomonas putida group in the NCBI taxabase.

Genome annotation was performed using NCBI’s Prokaryotic Genome Annotation Pipeline version 4.4 (13). Based on this analysis, a total of 4,572 genes were predicted, including 4,370 protein coding genes, 19 rRNA genes, 73 tRNA genes, 4 noncoding RNA (ncRNA) genes, and 106 pseudogenes.

Functional annotation performed with Rapid Annotations using Subsystems Technology (14–16) showed that most genes were associated with amino acid and derivative metabolism (617 genes) or carbohydrate metabolism (388 genes). In addition, 119 genes were associated with virulence, disease, and defense, with 95 specifically involved in resistance to antibiotics and toxic compounds. Lastly, 58 functional units associated with iron acquisition were found.

### Data availability.

The complete genome sequence of *Pseudomonas* sp. strain SGAir0191 has been deposited in DDBJ/EMBL/GenBank under accession number CP025035. Raw reads were deposited in the Sequence Read Archive (SRA) under accession numbers SRR8894400, SRR8894401, and SRR8894402.
